# Tumor-Derived Extracellular Vesicles: Modulation of Cellular Functional Dynamics in Tumor Microenvironment and Its Clinical Implications

**DOI:** 10.3389/fcell.2021.737449

**Published:** 2021-08-31

**Authors:** Nathalia Leal Santos, Silvina Odete Bustos, Darshak Bhatt, Roger Chammas, Luciana Nogueira de Sousa Andrade

**Affiliations:** ^1^Center for Translational Research in Oncology, Instituto do Câncer do Estado de São Paulo, Hospital das Clínicas da Faculdade de Medicina da Universidade de São Paulo, São Paulo, Brazil; ^2^Department of Medical Microbiology and Infection Prevention, University Medical Center Groningen, University of Groningen, Groningen, Netherlands

**Keywords:** extracellular vesicles, tumor microenvironment, cell communication, tumor progression, functional dynamics

## Abstract

Cancer can be described as a dynamic disease formed by malignant and stromal cells. The cellular interaction between these components in the tumor microenvironment (TME) dictates the development of the disease and can be mediated by extracellular vesicles secreted by tumor cells (TEVs). In this review, we summarize emerging findings about how TEVs modify important aspects of the disease like continuous tumor growth, induction of angiogenesis and metastasis establishment. We also discuss how these nanostructures can educate the immune infiltrating cells to generate an immunosuppressive environment that favors tumor progression. Furthermore, we offer our perspective on the path TEVs interfere in cancer treatment response and promote tumor recurrence, highlighting the need to understand the underlying mechanisms controlling TEVs secretion and cargo sorting. In addition, we discuss the clinical potential of TEVs as markers of cell state transitions including the acquisition of a treatment-resistant phenotype, and their potential as therapeutic targets for interventions such as the use of extracellular vesicle (EV) inhibitors to block their pro-tumoral activities. Some of the technical challenges for TEVs research and clinical use are also presented.

## Introduction

Malignant tumors are defined as a microenvironment composed not only by different clones of tumor cells, but also by stromal cells as well as extracellular matrix (ECM) (Quail and Joyce, 2013). All these components interact with each other dictating the natural history of the disease and the response to treatment. From an ecological point of view, these interactions can be described as cooperative or competitive and, in both cases, depend on the mechanism of cellular communication (Pelham et al., 2020). In fact, these interactions are dynamic and mediated not only by soluble factors secreted by cells or trapped in the ECM, but also by extracellular vesicles (EVs) (Tkach and Théry, 2016).

Extracellular vesicles are spherical lipid bilayer structures secreted by many cell types which play an important role in tumor progression (Schubert and Boutros, 2021). In the last decade, it has become clear that EVs shuttle messages between cells at short and large distances, changing the way cell communication has been described so far (Raposo and Stahl, 2019). Two major classes of EVs, exosomes (or small vesicles, from 30 to up 150 nm in size) and microvesicles (or large vesicles, from > 100 nm to 1 μm) are the most studied and better characterized among the other EVs types. Exosomes have endocytic origin, being formed as intraluminal vesicles (ILVs) by inward budding of the limiting membrane of late endosomes. After the fusion of multivesicular bodies with the plasma membrane, exosomes are released into the extracellular environment. By contrast, microvesicles are formed by outward budding of the plasma membrane (van Niel et al., 2018). The other classes of EVs, like large oncosomes, apoptotic bodies and platelet-derived vesicles, will not be discussed in this Review.

Amongst the diverse means of communication, EVs are the only ones that are known to carry almost all types of signaling molecules varying from DNA, different types of RNAs, protein/ligands, enzymes, metabolites, growth factors, lipids and even cytokines as recently described (Fitzgerald et al., 2018; Anand et al., 2019; Lázaro-Ibáñez et al., 2019; O’Brien et al., 2020). In general; EVs exert their effects by the transfer of their cargo to recipient cells modulating their phenotype and function (Yáñez-Mó et al., 2015). In this review, we will discuss some examples of how EVs can orchestrate cellular communication in the tumor microenvironment (TME) to promote tumor progression. In addition, we will exploit the role of these EVs in treatment response and discuss the gaps and future directions for clinics and EVs research. We apologize to all authors whose relevant and important work could not be cited due to space constraints.

## TEVs as Important Mediators of Cell Communication During Malignant Transformation and Tumor Progression

In the past years, several groups showed that extracellular vesicles secreted by tumor cells (Tumor–Derived Extracellular Vesicles, TEVs) can modulate the cancer hallmarks described in 2011 (Hanahan and Weinberg, 2011). For example, it is already known that TEVs are able to promote cooperation with stromal cells like endothelial cells to allow tumor development (Aslan et al., 2019), to suppress the anti-tumor immune response in cancer patients (Sharma et al., 2020), and to signal at distant sites to resident cells for the establishment of pre-metastatic niche (Costa-Silva et al., 2015).

**FIGURE 1:**
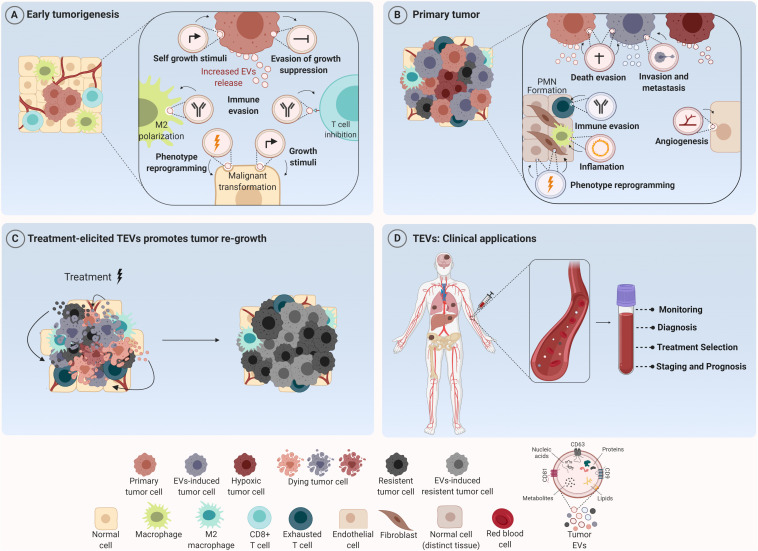
Tumor-derived extracellular vesicles (TEVs) as mediators of tumor development and their use as potential theranostic biomarkers in oncology. **(A)** TEVs have been recognized as important modulators of many biological processes that govern tumor initiation such as sustained cell growth, evasion of antiproliferative and death stimuli, and immune evasion. Their uptake by tumor and stromal cells induces phenotype reprogramming that supports malignant transformation and tumor establishment. **(B)** Along tumor progression these nanostructures are able to create a permissive microenvironment characterized by sustained angiogenesis, inflammatory and immunosuppressive milieu at short and long distances supporting the formation of the pre-metastatic niche (PNM). **(C)** In addition, TEVs secreted in response to therapy can elicit a bystander effect in both tumor and stromal cells, inducing cell survival and outgrowth resulting in tumor recurrence. **(D)** In the clinical setting, since TEVs can reach all body fluids, they can be found in peripheral circulation and used for cancer diagnosis, prognosis and also as biomarkers for monitoring tumor response to therapies. Figure created using Biorender.

In fact, TEVs can also participate in tumor initiation and propagation. In the study of Kalra et al. (2019), TEVs from colorectal cancer (CRC) cells harboring β-catenin mutation were shown to transfer this protein to wild-type CRC recipient cells, inducing the activation of WNT signaling pathway in these cells and, consequently, boosting tumor growth in xenograft models. Similarly, Fonseka et al. (2019) showed that TEVs secreted by N-myc-amplified neuroblastoma cells increased the proliferative and migratory potential of N-myc-non-amplified tumor cells, increasing tumor aggressiveness. Furthermore, very recently, an interesting study conducted by Kilinc et al. (2021) demonstrated that oncogene activation led to regulation of EVs release and cargo, suggesting that these nanostructures are biologically relevant even in the initial phases of the disease (Figure 1A).

Beyond this scenario, an important hallmark of solid tumors is the induction of angiogenesis – a *sine qua non-condition* for continuous tumor growth and progression (Folkman, 1975). More recently, several groups showed that TEVs are one of the mediators for this process (Yang et al., 2018; Bai et al., 2019; He et al., 2019; Wang et al., 2019; Shang et al., 2020; Biagioni et al., 2021). Indeed; in 2017, it was demonstrated that TEVs from glioma stem-like cells carried VEGF-A (Treps et al., 2017). One year later, Tang et al. (2018) observed that TEVs from ovarian cancer contained E-cadherin in their surface which were able to form heterodimers with VE-cadherin in endothelial cells, promoting their sprouting and angiogenesis *in vivo*. Furthermore, Sato et al. (2019) demonstrated that higher expression of EPHB2 within EVs isolated from head and neck squamous carcinoma cell lines were able to promote angiogenesis through the activation of ephrin reverse signaling in endothelial cells. Interestingly, Ko et al. (2019) observed that TEVs from ovarian cancer induced migration and tube formation by endothelial cells through a bevacizumab-insensitive VEGF presented in vesicles, showing that TEVs can also impair the efficacy of anti-angiogenic therapies. Furthermore, the pro-angiogenic role of TEVs can also be a consequence of their uptake by other stromal cells like fibroblasts, inducing a pro-tumoral phenotype in these cells (Zhou et al., 2018; Fan et al., 2020; Figure 1B).

Additionally, environmental stimuli like hypoxia can somehow modify TEVs release and/or TEVs cargo leading to increased angiogenesis (Chen X. et al., 2018; Guo et al., 2018; Wang et al., 2018; Park et al., 2019; Qian et al., 2020). Under hypoxia, for example, lung cancer cells produce more exosomes in comparison to normoxia. Elevated levels of miR-23a were found inside these exosomes which targeted prolyl hydroxylase 1 and 2 (PHD1 and 2), leading to an increase in HIF-1 alpha in endothelial recipient cells and sustained angiogenesis and tumor growth *in vivo*. Furthermore, enrichment of Wnt4 protein and carbonic anhydrase 9 (CA9) in TEVs in response to hypoxia were demonstrated to be responsible for increased angiogenesis in colorectal cancer (Horie et al., 2017; Huang and Feng, 2017). Beyond angiogenesis, TEVs secreted under hypoxia can also promote cancer progression through the induction of drug resistance (Dorayappan et al., 2018), stemness phenotype and increased invasive capability (Ramteke et al., 2015).

In fact, concerning metastasis, the mechanisms triggered by TEVs are quite diverse. EVs derived from the plasma of CRC patients were enriched with ITGβL1 and associated with lung and liver metastasis. This effect was caused by the activation of resident fibroblasts, which were induced to secret pro-inflammatory cytokines, promoting the establishment of the pre-metastatic niche (Ji et al., 2020). Similarly, EVs secreted by pancreatic tumor cells were shown to be selectively taken up by Kupffer cells (KC) in the liver leading to the release of TGFβ and the production of fibronectin by hepatic stellate cells, which recruited bone marrow-derived macrophages to establish a pro-inflammatory milieu to facilitate tumor metastasis (Costa-Silva et al., 2015). Few years later, Zeng et al. (2018) observed that TEVs from CRC not only increased vascular permeability, but also enhanced CRC metastasis in liver and lungs. Moreover, exosomes carrying miR-122 released by breast cancer cells reduced glucose uptake by normal recipient cells in pre-metastatic niche, increasing nutrient supply for metastatic cells Fong et al., 2015). Then, the metastatic effect by TEVs can also be mediated by their effect in stromal cells like fibroblasts (Fang et al., 2018; Xu et al., 2019); macrophages (Liang et al., 2019; Zhao et al., 2020) and in bone stroma (Dai et al., 2019) at long distances (Figure 1B).

## The Establishment of an Immunosuppressive Environment by TEVs

Regarding the regulation of immune cells in TME, TEVs can educate infiltrating immune cells to cooperate with malignant cells, creating a permissive environment for tumor progression. In this context, EVs shed by GBM cells displaying PD-L1 were able to induce CD8 T cells exhaustion by directly binding to PD-1, thus facilitating tumor progression and impairing immunotherapy treatment efficacy (Ricklefs et al., 2018). Similar results were obtained for EVs derived from metastatic melanoma (Chen G. et al., 2018). In NSCLC patients, PD-L1 enriched exosomes from these patients inhibited IL-2 and IFN-γ production by T CD8^+^ lymphocytes (Kim et al., 2019). In fact, exosomal PD-L1 is already pointed out as an important biomarker in treatment management which demonstrates the translation of these findings into clinical practice. In hepatocarcinoma cells, TEVs can induce an immunosuppressive phenotype also in infiltrating B cells (Ye et al., 2018). In addition, interestingly, these immunosuppressive effects of TEVs also occur at long distances beyond the primary tumor site. Hsieh et al. (2018) showed that EVs shed by head and neck cancer cells induced M2 polarization through the transfer of miR-21 to CD14^+^ human monocytes, favoring tumor growth (Figure 1B). In fact, this work shows that these cooperative relationships mediated by TEVs are not restricted to the tumor primary site and indeed can be established even at larger distances due to their presence in all body fluids, reinforcing the notion of cancer as a systemic disease under the influence of vesicles.

## Treatment-Elicited TEVs Promotes Tumor Re-Growth

Tumor recurrence is considered one of the major causes of treatment failure, being directly correlated with a poor prognosis. The cellular mechanisms behind this involve intrinsic and/or acquired resistance (Zhu et al., 2021). The latter one can be mediated by vesicular cargo transfer of multidrug resistance transporters (Bebawy et al., 2009; Corcoran et al., 2012), anti-apoptotic and pro-tumorigenic molecules (Khan et al., 2012; Vella et al., 2017) from resistant to sensitive tumor cells, for example. Mrowczynski et al. (2018) showed that EVs derived from nervous system cancer cells upon Ionizing Radiation (IR) therapy could induce cell death evasion and consequent treatment resistance. Interestingly, higher radiation doses were significantly correlated with more expressive decrease in tumor suppressive molecules (STAT4, TPM1, miR-516, and miR-365) and greater increase of oncogenic cargo (CCND1, ANXA2, NPM1, and miR-889). Also, vesicles derived from dying pancreatic cancer cells after radiotherapy were shown to be enriched with miR-194-5p and could potentiate the survival of recipient cells by up-regulating DNA damage responses. In addition, the inhibition of TEVs release by aspirin significantly suppressed tumor re-growth and increased the survival of tumor-bearing mice (Jiang et al., 2020).

Moreover, EVs released upon chemotherapy have also been reported to promote tumor resistance. Survivin was found to be enriched in EVs secreted by chemotherapy-treated breast cancer cells and was able to promote the survival of tumor cells and tumor associated fibroblasts exposed to paclitaxel (Kreger et al., 2016). Furthermore, targeted therapy treatment with vemurafenib in BRAF-mutated melanoma cells resulted in altered miRNA and protein profile within EVs, inducing increased resistance in recipient cells (Lunavat et al., 2017). In accordance with these findings, Marconi et al. (2021) demonstrated that TEVs secreted by ERBB2 + breast cancer cells in response to trastuzumab carry a different protein cargo that are known to be associated with cytokinesis, lipid metabolism and organelle organization, indicating that this process might be altered in the recipient cells.

Our group recently demonstrated that EVs release by melanoma cells treated with temozolomide are taken up not only by tumor cells, which showed an increase in pluripotent and DNA repair gene expression levels, but were also able to induce a M2-phenotype in macrophages, promoting tumor repopulation in nude mice (Andrade et al., 2019). Additionally, EVs released by breast cancer cells upon paclitaxel or doxorubicin treatment were reported to contain higher amounts of ANXA6 protein, which could induce the pre-metastatic niche formation, by promoting Ccl2 expression, monocyte expansion and NF-kβ-dependent endothelial cells activation in pulmonary tissues, favoring lung metastasis at *in vivo* models (Keklikoglou et al., 2019). In myelomas, TEVs released upon chemotherapy were able to induce ECM remodeling and promote chemoresistance and relapse (Bandari et al., 2018; Figure 1C).

Still under this context, another process which is often sped up and induced by cytotoxic therapy is the autophagic flux. In the last few years, some studies have shown cross-regulation between autophagy and exosome release. Initially, autophagy was known as a catabolic process of intracellular degradation of proteins and organelles destined to the recycling of material and the balance of energetic cellular metabolism maintaining cellular homeostasis. However, autophagy can also interfere within the TME communication through its secretory function called secretory autophagy (SA) in a similar way as observed for TEVs (Thorburn et al., 2009; Claude-Taupin et al., 2018; Rak, 2020). Based on studies showing the similarities between EVs and autophagy concerning their biogenesis and secretory function (Murrow et al., 2015; Galluzzi et al., 2017; Pathan et al., 2019), one might speculate that this interconnection can be used by tumor cells to establish a cooperative relationship among different cells in the TME, impacting both tumor progression and treatment response in some tumors as discussed below.

Few years ago, Dutta et al. (2014) observed that exosomes released by breast cancer cells were taken up by normal epithelial cells, which was accompanied by an increase in ROS levels and autophagy in recipient cells. Consequently, these cells secreted soluble growth factors that induced the proliferation of malignant cells. More recently, cooperation among different cells in TME was shown to be dependent on the synergism between these two secretory pathways. In response to oxidative stress, pancreatic ductal adenocarcinoma cells released exosomes enriched in KRAS^G12D^ during autophagy-dependent ferroptosis. These vesicles were engulfed by macrophages which, in turn, were polarized to M2 phenotype and promoted tumor growth in a mouse model (Dai E. et al., 2020).

About tumor response to therapy, it has been demonstrated that the use of chemotherapy and inhibitors of mTOR pathway led to an increased autophagic flux and, simultaneously, the exosome release (Hessvik et al., 2016; Xu J. et al., 2018; Ma et al., 2019). Exosomes harvested from irradiated brain cells carrying the miR-7 induced autophagy and transferred this signal to non-irradiated lung cells, mediating a bystander effect of autophagy in the lung after brain irradiation (Cai et al., 2017). In non-small cell lung carcinoma cells, exosomal miR-425-3p released in response to cisplatin decreased responsiveness to this drug via targeting the AKT1/mTOR signaling pathway and upregulation of autophagic activity in recipient cells. On the other hand, enhanced miR-567 levels in HER2 + breast cancer cells were packaged into exosomes which were responsible for suppressing autophagy and reversing chemoresistance by targeting ATG5 in recipient cells (Han et al., 2020).

## TEVs: From the Bench to the Bedside

As already discussed, TEVs are multifaceted regulators of tumor progression and response to different therapeutic modalities. Although some aspects regarding their biogenesis and cargo sorting are still largely unknown, their diagnostic and therapeutic potential in oncology have been explored with enthusiasm for several groups. At some level, TEVs cargo reflects the molecular composition of malignant cells and, since these nanostructures can be found in all body fluids, they can serve as circulating biomarkers in liquid biopsy (LeBleu and Kalluri, 2020). Moreover, many studies demonstrated that the level of EVs in plasma is significant higher in cancer patients than healthy individuals as reported for esophageal squamous cell carcinoma (Matsumoto et al., 2016) and glioblastoma (Osti et al., 2019), indicating that EVs plasma quantification can be a useful surrogate indicator for cancer screening. Besides that, it has been demonstrated that plasma exosomal level increases with tumor stage progression as observed in patients with non-small cell lung cancer (Liu et al., 2018) and can be used also as an indicator of disease progression (Figure 1D).

Another aspect of EVs biology that has been explored in translational studies relies on the presence of macromolecules carried by these nanostructures. A very recent study by Hoshino et al. (2020) identified tissue-specific and tumor derived proteins in TEVs from cancer plasma patients in comparison to health individuals, indicating the potential use of vesicular proteins in cancer diagnosis. Moreover, in colorectal patients (CRC), it was found that elevated levels of serum exosomal circ-PNN, a circular RNA, can be used for CRC diagnosis according to the validation analysis conducted by the authors (Xie et al., 2020). Interestingly, even the lipidic profile of plasma exosomes shows to be a promising biomarker in cancer (Bestard Escalas et al., 2021). To date, there are 16 clinical studies registered in Clinicaltrials.gov that aim to evaluate EVs potential in cancer diagnosis for different tumors, demonstrating that although the biology behind these nanostructures is still largely unknown, their use in clinical practice has been already explored.

Another promising use of TEVs in clinical settings is the potential of EVs as vehicles for the delivery of therapeutic agents which has also generated considerable excitement in the field. In fact, several studies evaluating the use of exosomes for the delivery of miRNAs, mRNAs, proteins, peptides, and synthetic drugs have been performed in the last years (Xu R. et al., 2018; Dai J. et al., 2020). Drugs such as doxorubicin (Srivastava et al., 2016), paclitaxel (Kim et al., 2016) and siRNA against oncogenic KRASG12D (Kamerkar et al., 2017) were successfully loaded in EVs and demonstrated potential anticancer effects *in vitro* and *in vivo*.

On the other hand, one alternative to explore the use of TEVs in cancer therapy is to reduce the exosome production or inhibit their secretion by tumor cells (Qi et al., 2016). A plenty of exosome inhibitors have been discovered and tested *in vitro* and in pre-clinical models to evaluate their effectiveness against transformed cells especially as neoadjuvant compounds. Most of them were developed to target important molecules of the exosome biogenesis machinery such as Rab27A and sphingomyelinases (Zhang et al., 2020) showing exciting results.

## Outstanding Questions, Gaps, and Conclusion

**FIGURE 2:**
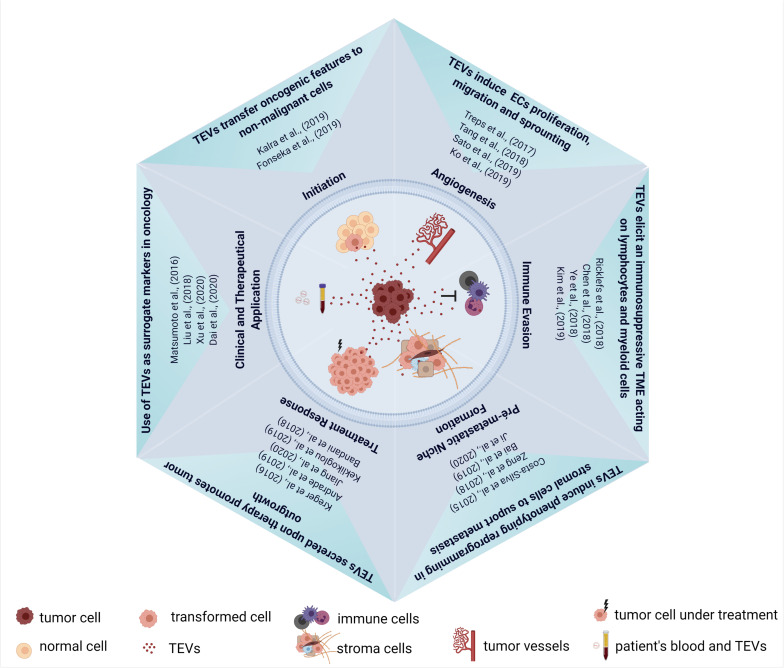
Schematic representation of TEVs in cancer biology. Upper corner: pro-tumorigenic effect of TEVs in tumor initiation and progression. Right corner: establishment of an immunosuppressive environment by TEVs. Lower corner: effect of TEVs in pre-metastatic niche formation and in tumor repopulation after therapy. Left corner: the use of TEVs as theranostic biomarkers in oncology. Some of the published articles illustrating these mains findings of TEVs were cited in the figure. Figure created using Biorender.

Although remarkable progress has been made in the EVs field, as summarized in Figure 2, there are some gaps in our understanding of molecular mechanisms that control vesicle packaging and also how the cargo loading can be modified in response to different stimuli such as cancer therapies. This aspect is crucial for the comprehension of how EVs can promote resistance and tumor recurrence after therapy and to design new therapeutic strategies to minimize and block these pro-tumoral effects. Furthermore, EVs heterogeneity should be considered in pre-clinical and clinical studies and our understanding of functional differences among EVs classes is still limited. The answer for this question in particular will be necessary especially for the use of EVs as biomarkers in cancer diagnosis and prognosis.

Second, technical challenges are still debated in the EVs community and an effort for the standardization of methods for EV isolation, purification, quantification, and molecular characterization has been made to allow interlaboratory comparisons of pre-clinical and clinical data.

Third, even though interventions in TEVs like the ones involving drug loading and the use of EVs secretion inhibitors indicate therapeutic potential, new criteria become relevant to be investigated such as the timing of therapy, tumors to be treated and chemo-radio-immunotherapy combinations. It is also crucial to determine how the effect of different strategies to regulate exosomes in particular could influence the autophagy machinery or vice versa. Nevertheless, beyond these considerations, we believe that the study of the complex regulation between both pathways after chemo and radiotherapy, among other modalities, may open avenues for the design of novel therapies as well as improve the current ones, avoiding tumor recurrence. Then, in our opinion, the comprehension of the tumor-driven cooperation mediated by TEVs during tumor progression and upon therapy will pave the way to improve therapy outcomes in cancer patients.

## Author Contributions

RC and LA conceptualized the manuscript, reviewed, and edited the manuscript before submission. LA, NS, SB, DB, and RC provided intellectual input, analyzed the literature, and participated in the writing. NS prepared the figures. All authors read and approved the final version of the manuscript.

## Conflict of Interest

The authors declare that the research was conducted in the absence of any commercial or financial relationships that could be construed as a potential conflict of interest.

## Publisher’s Note

All claims expressed in this article are solely those of the authors and do not necessarily represent those of their affiliated organizations, or those of the publisher, the editors and the reviewers. Any product that may be evaluated in this article, or claim that may be made by its manufacturer, is not guaranteed or endorsed by the publisher.
